# Sexual Motivation (Desire): Problems with Current Preclinical and Clinical Evaluations of Treatment Effects and a Solution

**DOI:** 10.3390/bs15050642

**Published:** 2025-05-09

**Authors:** Anders Ågmo

**Affiliations:** Department of Psychology, University of Tromsø, 9037 Tromsø, Norway; anders.agmo@uit.no; Tel.: +47-77646365

**Keywords:** low sexual interest/arousal disorder, preclinical drug tests, clinical trials, implicit motivation, genital responses

## Abstract

There has been an extensive search for efficient pharmacological treatment of female sexual interest/arousal disorder and other sexual dysfunctions. However, available treatments have met limited success, except for the drugs used for treating erectile deficiency. A possible reason for this may be that both the preclinical and clinical evaluation of treatment effects have been inadequate. The present literature review shows that the intensity of sexual approach behaviors in non-human animals appears to be predictive of clinical effect whereas the traditional studies of copulatory behaviors and associated motor patterns have questionable predictive power regarding effects on human sexual desire. In clinical studies, it is essential to include the unconscious components of sexual motivation in any approach to its quantification. This basic fact is incompatible with the use of self-reports for evaluating treatment effects on motivation. Genital responses to sexual incentives are automatic and therefore outside of volitional control and can, therefore, provide unbiased estimates of the intensity of sexual motivation. These responses may be objectively quantified. Tests for implicit sexual motivation must also be used for capturing unconscious mental components. Including the unconscious components of sexual motivation as well as of objective measures of genital responses in clinical studies may improve evaluations of the effectiveness of drug treatment of low sexual interest/arousal disorder. In preclinical studies, predictive validity can be improved by quantifying sexual approach behaviors rather than copulatory behavior. The paradigm shift suggested here may finally allow for the discovery of efficient treatments for some sexual dysfunctions.

## 1. Introduction

Sexual satisfaction is important for wellbeing (Anderson, 2013; Lewis et al., 2024; Mitchell et al., 2021) and contributes significantly to the quality of life (Blanchflower & Oswald, 2004; Cheng & Smyth, 2015; Kashdan et al., 2018; Stephenson et al., 2024). Consequently, lack of a satisfactory sex life contributes to illbeing and reduced quality of life (Flynn et al., 2016). Sexual dysfunctions of all kinds tend to reduce sexual satisfaction, hence quality of life (Gezginci et al., 2024; Hisasue et al., 2005). It is no wonder, then, that much effort is and has been invested in the search for efficient pharmacological treatments of such dysfunctions. This search may involve exploratory and preclinical studies in non-human animals, mainly rodents.

In the absence of known sexual dysfunctions in rats, mice, and hamsters and in other species commonly used in the laboratory, scientists have either evaluated drug-induced changes in copulatory behavior in a random sample of animals or in a sample selected according to some criterion. The rationale for the former kind of studies is that altered copulatory behavior in animals would translate into similar alterations in humans with a sexual dysfunction. The latter approach is based on the notion that the interindividual variation in sex behavior may provide the basis for selecting animals displaying some characteristic of a human sexual dysfunction. Rats with an unusually short ejaculation latency are often used in studies of premature ejaculation (Clement et al., 2012; Gao et al., 2023; Huang et al., 2023), whereas females spending little time with males in a pacing cage have been suggested to be a model of low sexual interest in women (Snoeren et al., 2011a), just to give two examples. However, rats having short ejaculation latencies cannot be given the diagnosis of premature ejaculation. Ejaculation latency has been found to have a lognormal distribution in humans (Janssen & Waldinger, 2016), and it can be assumed that this is the case also in rats. This means that short ejaculation latency is normal for a certain proportion of rats. Likewise, the time female rats spend with males in a pacing procedure has an approximate normal distribution, meaning that a certain proportion of female rats always will spend little time with males. Even though we might accept the face validity of such selection procedures, there is an important issue with them: mere alterations in sexual behavior are not enough for being diagnosed with a sexual dysfunction. According to the DSM-5-TR (American Psychiatric Association, 2022) as well as the ICD-11 (World Health Organization, 2024), long-lasting clinically significant distress is one of the requisites for the diagnosis of sexual dysfunction. Whether fast ejaculation or spending little time in the male’s compartment in a pacing test cause distress in male or female rats is not known.

In the present communication, I will argue against the predictive validity of preclinical procedures based on evaluation of copulatory behavior. First, however, the few drugs approved for the treatment of human sexual dysfunctions will be briefly described. Both clinical and preclinical observations will be mentioned. Then, I will present arguments showing that the mechanisms determining the behavioral manifestations of sexual motivation in rodents and other non-human animals are fundamentally different from those operating in humans even though the endocrine and neurobiological bases might be similar (Ågmo, 2024). Whereas the behavioral expressions of sexual motivation are mostly automatic in non-human animals, the automatic (unconscious) processes are heavily influenced by conscious, volitional acts in humans. To substantiate this claim, I will briefly describe a model of sexual motivation and specify how unconscious (implicit) as well as conscious processes determine the manifestation of this motivation in humans and how the unconscious contributions can be revealed.

Another issue to be discussed is whether the evaluations of clinical efficacity of drug treatment are flawed because they rely on self-reports. The inherent weaknesses in human studies based on self-reported sexual motivation and the number of satisfactory sexual events per unit time will be discussed. I will then suggest that implicit measures of sexual motivation may provide more accurate estimations than self-reports do. One consequence of the heavy reliance on self-reports is that the guidelines for evaluating the effects of potential treatments of low sexual interest or arousal established by the US Food and Drug Administration may lead to false conclusions. As a result, data from both phase III and phase IV studies may fail to reflect the real efficacity of any treatment. Considering the questionable predictive validity of the common animal models combined with the flawed evaluation of effects in humans, the lack of progress in the pharmacological treatment of sexual disorders other than erection becomes understandable. The urgent need for a paradigm change both in preclinical and clinical studies should have become evident.

## 2. Drugs Approved for Treatment of Sexual Dysfunctions: Clinical Data

Sometimes, the efforts to find efficient pharmacological treatments of sexual dysfunctions have been successful. Erectile deficiency can be treated with phosphodiesterase-5 inhibitors, and more than 70% of treated men report substantial improvement (Palumbo et al., 2001). It may be worthwhile to note that the proerectile drugs act locally in erectile tissue. All the approved phosphodiesterase-5 inhibitors have been commercial successes, especially the first one, sildenafil (commercial name Viagra^®^). The break-through discovery of sildenafil as a proerectile compound was based on clinical observations (Goldstein et al., 2019), with no contribution from behavioral studies in non-human animals.

Other sexual disorders have fared less well. A drug thought to be helpful in the treatment of early ejaculation, dapoxetine, is a short-acting inhibitor of serotonin (5-hydroxytryptamine) reuptake with minimal actions on dopamine and noradrenaline (Modi et al., 2006). It is taken orally at least one hour before expected sexual activity. Dapoxetine has been registered in the European Union and many other countries, but few men have chosen to continue using the drug, mainly because of the high cost and disappointment with the most limited effect (Mondaini et al., 2013; Park et al., 2017). It has not been registered in the USA. It is not evident that dapoxetine is superior to psychotherapeutic treatments, according to a recent review (Pizzol et al., 2023).

Female sexual interest or arousal disorder (formerly hypoactive sexual desire disorder) has inspired much research, both preclinical and clinical. Before entering into a discussion of this research, it may be convenient to address the issue of terminology. It is not customary to use the vernacular term “sexual desire” in non-human animals. Instead, the scientific term “sexual motivation” is used. An etymological analysis of the word desire tells us that it has several meanings. According to the Merriam-Webster online dictionary they are: (1) conscious impulse toward something that promises enjoyment or satisfaction in its attainment; (2) sexual urge or appetite; (3) something longed or hoped for; and (4) a usually formal request or petition for some action (https://www.merriam-webster.com/dictionary/desire, accessed on 24 April 2025). The American Psychological Association defines motivation in the following way: The impetus that gives purpose or direction to behavior and operates in humans at a conscious or unconscious level (https://dictionary.apa.org/motivation, accessed on 24 April 2025). The main difference between the regular dictionary definition of desire and the APA definition of motivation is that desire is conscious whereas motivation may be unconscious. This is of utmost importance, because the clinical term “sexual desire” may exclude unconscious elements making it clearly different from the scientific term “sexual motivation”, which includes both conscious and unconscious components. Consequently, I use the term desire when referring only to the conscious components of motivation. Otherwise, the latter term is used. An analysis of the theoretical implications of the complex relationship between desire and motivation is beyond the scope of the present contribution. However, excellent reviews are available (Lucas & Nieto, 2023; Nimbi et al., 2020).

Two drugs for treating female sexual interest or arousal disorder have been approved in the USA. The first, flibanserin, was approved by the Food and Drug Administration in 2015. The drug is a 5-hydroxytryptamine (5-HT) 1_A_ receptor agonist and 5-HT_2A_ antagonist with downstream effects on dopaminergic and noradrenergic neurons (Stahl et al., 2011). It is taken daily as a bedtime tablet. The second, bremelanotide, is an agonist at melanocortin 3 and 4 receptors (Yuan & Tao, 2022). It was approved in the USA in 2019. This drug is njected subcutaneously in the abdomen or thigh some 45 min before intended sexual activity. The clinical importance of these drugs is a matter of debate. Even though the effect may be superior to placebo, it is small (Cipriani et al., 2023; Davis, 2024; Spielmans, 2021; Spielmans & Ellefson, 2024). For example, a recent metanalysis found that flibanserin enhanced the number of satisfactory sexual events per month with only 0.69 in premenopausal women and with 0.37 in postmenopausal women (Kamrul-Hasan et al., 2024). This increase seems quite modest especially when considering that the notion of “satisfactory sexual event” is poorly defined (Kingsberg & Althof, 2011). Women are simply asked to record their sexual experiences as satisfying or not by paper or electronic diary. The affective response of satisfaction is not further specified. Even an extensive review paper (Pyke & Clayton, 2016) fails to provide a definition, probably assuming that an intuitive understanding of the term is sufficient. In Europe, there is no registered drug treatment for sexual interest or arousal disorder.

Genital responses to sexual stimuli are considered to be exquisite indicators of the intensity of sexual motivation (Ågmo & Laan, 2023). There are no published data concerning the effects of flibanserin on these responses. Bremelanotide has been found to lack effect on vaginal responses to sexual stimuli in women diagnosed with sexual arousal disorder (Diamond et al., 2006). To the contrary, the drug improved erection in response to unspecified visual sexual stimuli in men with erectile dysfunction (Diamond et al., 2004). The data on genital responses could be interpreted as showing that bremelanotide enhances sexual motivation in men whereas there is no such effect in women. Results from a recent clinical study with 21 male subjects suggest that this drug indeed may improve erectile function and enhances sexual desire in men suffering from sexual dysfunctions (Goldstein & Goldstein, 2024). Whether this effect is of clinical relevance remains to be established.

In contrast to the questionable efficiency of drug treatment of low sexual interest disorder as well as of early ejaculation, some psychotherapeutic approaches may be successful (Frühauf et al., 2013; Mestre-Bach et al., 2022; Slavin et al., 2020). The effect size reported in evaluations of cognitive behavior therapy and mindfulness meditation training has been found to be superior to the effect size found in studies with flibanserin and bremelanotide when corrected for effect size obtained in patients on the waiting list and during placebo, respectively (Pyke & Clayton, 2018). The difference, expressed as Cohen’s d, was 0.95 for psychotherapy vs. 0.45 for drug treatment. Not surprisingly, proponents of pharmacological treatment of female sexual interest/arousal disorder disagree (Kingsberg et al., 2021; Pfaus, 2022).

Due to the limited availability of approved treatments for low sexual interest/arousal disorder, some women recur to self-medication. Among the compounds used are l-arginine, ginseng, Ginko, and maca (a root vegetable grown in Peru) (Dording & Sangermano, 2018). Clear evidence for an effect superior to placebo of these products on sexual functions is lacking. Nevertheless, a study performed more than 20 years ago found that the combined treatment with yohimbine and l-arginine enhanced the vaginal response to a pornographic movie segment in women diagnosed with sexual arousal disorder. Neither yohimbine alone nor l-arginine alone had any effect (Meston & Worcel, 2002). As with so many other promising studies with a small number of participants, the results of this study have not been replicated. A review of the effects of l-arginine combined with different compounds was unconclusive (Cieri-Hutcherson et al., 2021). There are no published reports concerning the use of common recreational drugs for treating sexual dysfunctions. However, the sexual effects of drugs like ecstasy, psilocybin, cocaine and alcohol, just to mention a few, are obscure even in healthy individuals (Ågmo, 2015). There is not much reason to believe that they would be effective for treating sexual dysfunction.

## 3. Drugs Approved for Treatment of Sexual Dysfunctions: Preclinical Data

In the case of sildenafil, human tissue samples were used for discovering the mechanisms of the already observed proerectile responses in men (Boolell et al., 1996), sometimes using non-human tissue for confirmation (Wallis et al., 1999). Thus, the preclinical data were not used for making predictions about clinical efficacy, but to explain the already established clinical usefulness.

A few studies have evaluated the behavioral effects of dapoxetine in rodents. Data reported in a meeting abstract (Gengo et al., 2005) showed that dapoxetine indeed increased ejaculation latency after subcutaneous or oral administration. Whether other parameters of copulatory behavior were affected or not is not mentioned. However, a later study reporting complete data revealed that all parameters of copulatory behavior were altered (Huang et al., 2023), indicating a general impairment and slowing of behavior rather than a specific effect on ejaculation latency. Other reports show enhanced mount and intromission latency in addition to the increase in ejaculation latency (Gao et al., 2023; Zhu et al., 2022), again suggesting a general slowing of behavior. If these data can be generalized to men, it is no wonder that the drug has had modest clinical success.

Flibanserin has been found to increase paracopulatory behaviors in female rats (Gelez et al., 2013; Pfaus et al., 2004). Bremelanotide has been shown to have similar actions (Pfaus et al., 2007). The paracopulatory behaviors (sometimes called proceptivity) are stereotyped movements displayed immediately before and during copulatory interactions (Bergheim et al., 2015). Prominent among these behavior patterns are ear-wiggling (fast lateral head movements making the ears appear to quiver) and hop-darting (rapid hops with rigid legs combined with darting movements away from the male). Most hop-darts end with a presenting posture in which the female stands still while pressing the ventral body area against the floor with slightly spread limbs and elevated rump. The paracopulatory behaviors are thought to express sexual motivation (Beach, 1976), but direct support for that notion is lacking.

Pharmacological treatments aimed at increasing sexual motivation are believed to do so by enhancing the rewarding value of sexual activity. The basis for this belief is the hypothesis that low sexual interest or arousal is a consequence of low reactivity in the sexual reward systems (Arnow et al., 2009; Stahl, 2015). Based on this notion, a study in female hamsters evaluated the effects of bremelanotide on sexual reward (Borland et al., 2025). The conditioned place preference procedure was used. Briefly, this procedure consists of associating one distinctive environment with a rewarding event and another environment with a neutral event. After repeated pairings, the subjects are allowed to choose between the environments. The time spent in the rewarded environment is compared to the time spent in the non-rewarded compartment. The larger the difference, the larger the reward (Rossi & Reid, 1976). In the Borland et al. (2025) study, some females received bremelanotide before sexual interaction with a male in a distinctive environment while others were given saline. At a post-conditioning test, the drug-treated females were not different from control, showing that bremelanotide did not enhance the reward value of sex. It is noteworthy that copulatory behavior was unaltered in the drug-treated females. The lack of effect on sexual reward in female hamsters coincides with earlier observations in rats where bremelanotide also failed to affect sexual reward (Pfaus et al., 2004). However, the lack of effect on hamster copulatory behavior contradicts the stimulatory effects reported in rats.

In the case of flibanserin and bremelanotide the preclinical evidence for effects on sexual motivation is mixed, at best. In view of this, the questionable clinical utility and commercial failure shown by both drugs should not be surprising.

## 4. Conceptual Frameworks for Understanding Sexual Motivation

The sexual incentive motivation model (Ågmo, 1999; Ågmo & Laan, 2023; Toates, 2009, 2022; Touraille & Ågmo, 2024) has become dominant among contemporary approaches to sexual motivation. Basically, the model maintains that sexual motivation is activated by a sexually relevant incentive stimulus or, in humans, also by mental representation of such a stimulus. Provided that the central nervous system is in an appropriate state, the incentive stimulus will activate neural processes leading to visceral responses as well as to approach towards the incentive stimulus. The ensemble of these neural processes is labelled the sexual central motive state. Once activated, that state will lead to a series of visceral responses, like enhanced heart rate and blood pressure, increased respiration frequency, and release of some hormones. The most notable of the visceral responses is probably the increase in genital blood flow, leading to penile tumescence in men and clitoral engorgement as well as vaginal lubrication in women. The visceral responses are automatic, non-volitional reflexes outside of consciousness (Janssen et al., 2000; Laan & Everaerd, 1995). The magnitude of these responses depends directly on the level of activity in the sexual central motive state.

Another consequence of activity in the sexual central motive state is the potential display of approach behaviors. These behaviors involve activity in skeletal muscles and are therefore volitional and under conscious control. Cognitive evaluation of the appropriateness of the context for sexual approach is performed before any behavior is manifested. In addition, humans must assure that the emitter of the stimulus consents to be approached and subsequently consents to genital interaction (Ågmo & Laan, 2024).

During genital interaction with others, be it in the form of penile–vaginal intercourse, oral, or anal sex, the genitals of at least one of the participants will receive tactile stimulation. That stimulation feeds back to the sexual central motive state, further enhancing activity in it, until the threshold for activation of orgasm is passed. The same feedback operates also during solitary sex (masturbation).

On rare occasions, humans may approach and interact genitally with someone without having obtained consent. There are reports showing that women (Chadwick et al., 2022; Levin & van Berlo, 2004) as well as men (Bullock & Beckson, 2011) may experience orgasm during forced sexual interaction. This is entirely in agreement with the motivational model presented here. The basis is the automatic (inborn) connection between mechanical stimulation of the genitals and the sexual central motive state. Sensory feedback will not only produce pleasurable emotions, but it will also enhance activity in the sexual central motive state until orgasm is activated. This response is outside of volitional control, making it possible to obtain orgasm even with forced sexual interaction. This fact has been known for some time. Already in the 4th century, saint Augustine of Hippo recognized that raped women could experience orgasm against their will (Webb, 2013). Intense activity in the sexual central motive state caused by genital stimulation may well lead to orgasm even though the conscious evaluation of the context makes it highly aversive. This, in no way, impedes the genital vascular response to sexual stimuli from being a sensitive indicator of the level of sexual motivation.

To date, scientific research has found no evidence of consent having meaning in non-human animals. This concept is used in moral, political, and legal philosophy describing an act of permitting something to be done or of recognizing some authority. The fascinating discussion of the complexities of the notion of consent published some years ago by Heidi Hurd (Hurd, 1996) convincingly illustrates that it cannot be applied to actions displayed by non-human animals. Perhaps it is not even necessary, since most animals are sexually attractive only when ready to copulate (Le Moëne & Ågmo, 2018). Female rats, for example, attract males only during the period of behavioral estrus, i.e., the period when they respond with lordosis to every mount from a male (Chu & Ågmo, 2015a, 2014; Le Moëne et al., 2020). Lordosis in female rats is a behavior pattern consisting of a concave arching of the back with raised head and rump, stretched hind legs and the tail moved to one side (Pfaff & Lewis, 1974), thereby exposing the vaginal orifice making penile insertion possible. Similarly, many female primates attract males only when the sexual skin is swollen and brightly colored. These signs are estrogen-dependent and consequently associated with sexual receptivity (Bielert & van der Walt, 1982; Dixson, 1983).

In partnered as well as solitary sex in humans, genital stimulation usually ends when orgasm is achieved. In heterosexual couples, this often means that sex ends after the man’s ejaculation, regardless of whether the woman reached orgasm or not. The reason is the detumescence following ejaculation. The lack of erection impedes the continuation of coitus. In male non-human primates, the mechanisms underlying the cessation of sexual activity are poorly known (Dewsbury & Pierce, 1989). This is also the case in other animals. Male rats, for example, display multiple ejaculations before terminating a sexual encounter (Beach & Jordan, 1956). The behavior pattern preceding cessation of sexual activity may be either a mount, a vaginal penetration without ejaculation, or a vaginal penetration with ejaculation (Chu & Ågmo, 2015b).

In women, orgasm is not necessarily associated with cessation of sexual activity. Many women are multiorgasmic and may experience as many as 20 or more orgasms in rapid succession (Cerwenka et al., 2021; Darling et al., 1991; Kinsey et al., 1953). Female rats copulate continuously as long as the brain is exposed to appropriate concentrations of ovarian hormones (Chu & Ågmo, 2014). Regardless of the endpoint, the emotional consequences of the sexual interaction feed back to the incentive stimulus in the way that positive emotions enhance the incentive value of it whereas negative emotions reduce the incentive value. This feedback occurs both in humans and non-humans. It has even been proposed that lack of the expected positive affect produced by genital interaction and orgasm as well as possible aversive consequences of sex may underly disorders of reduced sexual desire (Ågmo & Laan, 2023). Continued absence of orgasm may reduce the incentive value of the partner (Dienberg et al., 2023; Leonhardt et al., 2018), for example. Likewise, feelings of guilt or shame following the sexual act as well as postcoital dysphoria may transform sexual incentives to stimuli predicting negative affect (Cado & Leitenberg, 1990; Emmers-Sommer et al., 2018; Maczkowiack & Schweitzer, 2019; Schweitzer et al., 2015). To the contrary, particularly intense postcoital positive affect may lead to enhanced incentive value of the stimuli present, and if systematically repeated, to exaggerated sexual activity. Thus, conscious emotional experiences associated with sexual acts may have profound consequences for future sexual behavior. In rodents, it has been found that associating ejaculation in the male with an aversive event devalues the female from a sexual to a neutral incentive, and the male will no longer display copulatory behavior when exposed to her (Ågmo, 2002).

**Figure 1:**
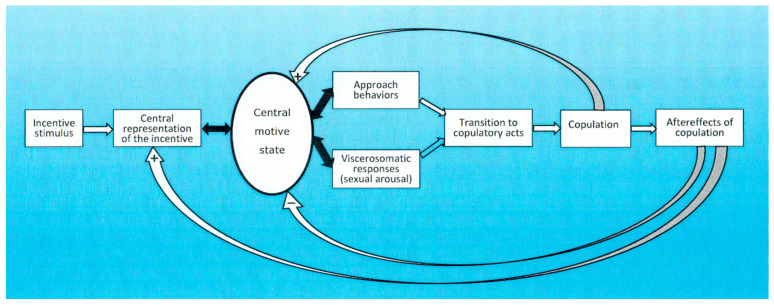
Schematic representation of the incentive motivation model. Empty arrows show unidirectional relationships, whereas filled arrows illustrate reciprocal relationships. The curved arrows represent feedback systems. For further details, see text. +, excitation. −, inhibition. Modified from Ågmo and Laan (2023) and reprinted under a Creative Commons license BY 4.0 (http://creativecommons.org/licenses/by/4.0/).

A schematic illustration of the sequence of events constituting a sexual encounter is shown in Figure 1.

The model outlined here is applicable to humans and other animals, as already mentioned. However, there are fundamental differences besides the already mentioned need for consent, exclusive to humans. One of these differences is that human sexuality is heavily influenced by social norms (Gagnon & Simon, 2002). There is currently no evidence for any social norms affecting sexual behavior in non-human animals. Whether such norms eventually will be discovered is an open question, but the possibility cannot be ignored (see Westra et al. (2024) for a discussion). The human norms are internalized in the form of scripts that humans are expected to follow during sexual encounters (Gagnon, 1990; Wiederman, 2015). The result is that almost every act displayed by humans during a sexual encounter has been subjected to cognitive evaluation. In other animals, notably rodents, sexual interactions can be interpreted and understood as a stereotyped sequence of viscerosomatic reflexes (Dewsbury, 1972; Le Moëne & Ågmo, 2019). Non-human primates are considered to display a somewhat less stereotyped sexual behavior than other mammals (Dewsbury & Pierce, 1989), but the variety of behavior patterns is infinitesimal compared to humans.

The behavior patterns displayed by humans during a sexual encounter are mostly volitional, i.e., under conscious control of the will. Other elements of the sexual act, like enhanced genital blood flow and consequent penile and clitoral engorgement as well as ejaculation and the experience of orgasm are non-volitional, controlled by the autonomic nervous system. The contractions of skeletal muscles accompanying orgasm in men and women are reflex responses (Bohlen et al., 1980, 1982; Shafik et al., 2009; Yang & Bradley, 2000), outside the control of the will. It could be stated that the consequences of genital stimulation are reflexive and automatic whereas the ways in which this stimulation is generated are purely volitional. The enormous variation in the motor acts performed during a human sexual encounter is illustrated in the vast choice of pornographic movies. Some focus on penile–vaginal intercourse, others on anal penetration or on different kinds of oral sex, still others on the employment of the most exotic sex toys. Not to mention the behaviors included under the label bondage, discipline, dominance, submission, and sadomasochism (BDSM) (Turley & Butt, 2015).

In most non-human animals, among those rats, mice, and hamsters, the copulatory acts are essentially automatic with no or slight involvement of volitions. This means that there is an abysmal difference between humans and most other animals with regard to the degree of conscious control of copulatory behavior patterns. This only applies to the behavioral manifestations of the activity in the sexual central motive state involving striated muscles. The autonomic and endocrine responses caused by activity in that state are outside of conscious control in all animals, including humans.

## 5. Unconscious and Conscious Processes

**Figure 2:**
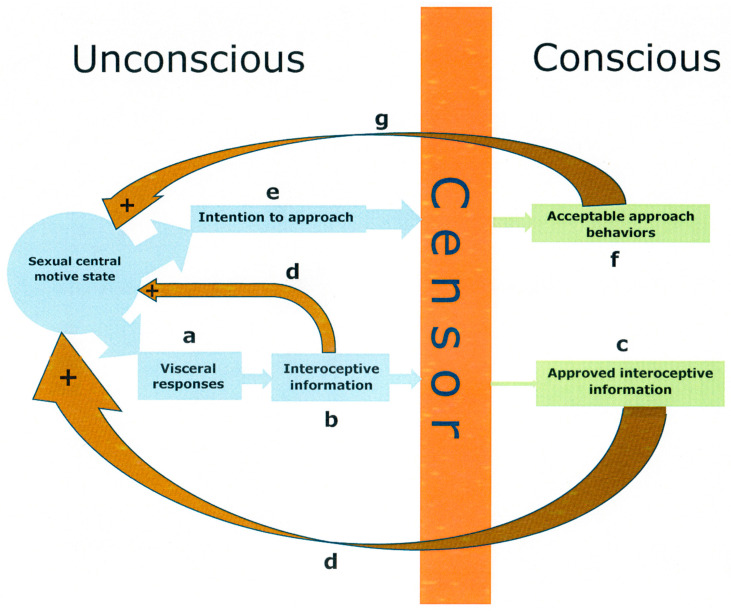
Illustration of the sequence of responses to activity in the sexual central motive state when exposed to a sexual incentive stimulus. The unconscious visceral responses activated ((**a**) in the figure), such as release of some hormones, increase of blood pressure and heart rate as well as altered psychogalvanic responses, clitoral engorgement and vaginal lubrication in women, and relaxation of smooth muscle in the cavernous bodies in the penis leading to erection in men, give rise to interoceptive information (**b**). Part of this information may reach consciousness (**c**), provided approval by the censor. Both the unconscious and conscious parts of the interoceptive information exert positive feedback on the sexual central motive state (**d**), further increasing its activity. The active motive state also generates unconscious or preconscious intentions to approach the emitter of the incentive stimulus (**e**). The censor determines whether these intentions are acceptable to social norms, and those that are deemed to be so may be manifested in behavior (**f**). The execution of these behaviors as well as additional stimuli generated by the approached incentive further enhance activity in the sexual central motive state via a positive feedback arch (**g**).

In his very influential description of the nature of the human mind, Freud made extensive use of the notion of the unconscious. Within the unconscious domain, motives of all kinds are found, among those the sexual motive (Westen, 1998). As long as a motive remains unconscious, it can have no influence on behavior, except for simple reflexes (Freud, 1915). The access to consciousness is controlled by the censor, a mechanism that tries to assure that motives enter consciousness only in a form that is socially acceptable, satisfactory to the superego (Freud, 1917). Inappropriate motives are impeded from reaching consciousness or are altered into innocuous motives. Likewise, the censor may block some interoceptive information from reaching consciousness—for example, the degree of vaginal lubrication. Once having reached consciousness, motives can determine behavior. This strongly simplified account is well suited for explaining the functioning of the sexual central motive state. The activity of that state is unconscious, but nevertheless it can stimulate genital blood flow, a simple reflex involving the autonomic nervous system. Other autonomous as well as endocrine responses can also be activated. There is no volitional control involved in these responses. A schematic representation of the unconscious and conscious responses activated by the sexual central motive state in the presence of sexual incentive stimuli is shown in Figure 2.

A note on terminology: The term unconscious was avoided by psychologists for many years, mostly for marking distance from psychoanalytic theory. It became customary to replace it by implicit (Kihlstrom, 2019). However, the term unconscious is regaining popularity (Greenwald & Banaji, 2017). Whether implicit is synonymous with unconscious is a matter of debate (Gawronski, 2009). Nevertheless, I use the term implicit when referring to authors using that term themselves. Elsewhere, I prefer unconscious.

Another important question with regard to the participation of consciousness in sexual motivation is whether sexual incentive stimuli must reach awareness for being able to stimulate the sexual central motive state. There is evidence suggesting that this is not the case. Subliminal presentation of sexual incentives has been found to activate the brain regions responding to supraliminal stimuli (Childress et al., 2008; Gillath & Canterberry, 2012; Gillath & Collins, 2016; Oei et al., 2014, 2012). These regions include the ventral striatum, dorsal anterior cingulate, amygdala, orbitofrontal cortex, anterior insula, hypothalamus, and midbrain tegmentum (Stoléru et al., 2012). More important, an elegant study revealed that repeated subliminal presentation of a neutral stimulus (conditioned stimulus) immediately before a pornographic movie fragment (efficient stimulus) made young men and women respond with genital engorgement when only the conditioned stimulus was presented (Hoffmann et al., 2004). The presentation was subliminal also at the test. Furthermore, conditioning with the subliminal stimulus was as efficient as supraliminal conditioning with the same stimulus. The fact that the subliminal (unconscious) stimulus activated a genital response confirms the proposal that there is an automatic although indirect connection between sexual incentives and the genital response (Janssen et al., 2000).

## 6. The Unconscious Made Conscious

Even though the control of the genital response to sexual incentives is unconscious and nonvolitional, the response itself may easily become conscious. Afferent nerves from the penis and vulva carry sensory information from the genitals to the spinal cord and to several supraspinal centers (Hubscher & Johnson, 1996; Lam et al., 2023; Tajkarimi & Burnett, 2011). It has been suggested that the sensory information from the penis is more accurate than that from the vulva, making it easier for men to estimate their level of sexual arousal and enhancing the concordance between self-reported sexual arousal and objectively measured magnitude of genital response (Laan & Janssen, 2007). The fact that concordance is higher in men than in women is well established (Chivers et al., 2010). Regardless of this, feedback from the genitals is thought to contribute to enhanced activity in the central motive state.

The preceding short summary of the contribution of conscious processes to the incentive-induced activation of sexual motivation is probably applicable only to humans. In the case of other animals, there does not seem to be any point in trying to distinguish conscious from unconscious processes. The conclusion to draw from this summary is that in humans, sexual incentive stimuli may activate sexual motivation (the sexual central motive state) without reaching consciousness. This activation is evidenced by the automatic visceral responses produced by such stimuli. Some of the visceral responses generate interoceptive information that enters conscious experience and further enhances the activity of the central motive state. Finally, the mechanical stimulation of the genitals during coitus enters consciousness and continues to excite the central motive state until orgasm is experienced.

Individuals diagnosed with sexual interest/arousal disorder show a genital response to sexual incentives indistinguishable from that in healthy individuals when tested in the laboratory (Blumenstock et al., 2024; Heiman et al., 2011; Sarin et al., 2014, 2016). This indicates that the dysfunction in most patients is situational, i.e., specific for a certain context or for a certain partner, rather than generalized. If the latter would be the case, then also the lab response to sexual incentives would be reduced. This means that some particular stimulus or stimuli must have lost their sexual incentive properties. In that case, cognitive behavior therapy may alter the cognitive evaluation of the affected stimuli and restore their sexual inventive properties. Alternatively, it can be maintained that the intact genital response shows that the sexual central motive state is as active as in healthy individuals, whereas the conscious manifestations of that activity have been suppressed. Therefore, there are no self-reported feelings of desire and no display of approach behavior. In that case, cognitive behavior therapy or mindfulness training could be helpful for restoring the access to consciousness of the activity in the sexual central motive state and the ensuing feeling of desire. Drugs could enhance the responsivity of the sexual central motive state and make the activity therein intense enough for trespassing the censor. Such an action might be particularly effective for weak incentive stimuli. In the rare cases of generalized sexual interest/arousal disorder, a state in which the sexual central motive state is unresponsive to all sexual incentives, drug treatment is probably the only option. It is possible that drug treatment is needed also for orgasmic disorder, sine the absence of orgasm suggests that the sexual central motive state never reaches the level of excitation needed for orgasm. It is likely that drug actions initially are unconscious since the activity of the sexual central motive state is outside of consciousness. However, some of the unconsciously initiated actions may become conscious—for example, genital engorgement and heightened heart rate and blood pressure as already mentioned many times.

The preceding account of the possible mechanisms involved in the origin of sexual arousal/interest disorder and their relationship to possible treatments is somewhat speculative. However, the basis for the speculations, that women diagnosed with this disorder show undiminished genital responses, is firmly established. There are not many well-founded explanations available, meaning that the proposals made above may fill an important void.

## 7. Quantification of Sexual Motivation: Humans

Sexual motivation itself, i.e., activity in the sexual central motive state, never enter consciousness whereas several of the manifestations of that activity become conscious. Sexual motivation per se cannot be quantified, but both the unconscious and conscious manifestations can be used for estimations. Conscious manifestations can be altered by volitions and are therefore not unbiased estimators. To the contrary, unconscious (automatic) manifestations provide fair estimates.

The study of the vascular genital responses manifested as erection and vaginal engorgement and lubrication has been of enormous importance for elucidating human sexual motivation, unmodified by conscious processes (Janssen & Prause, 2016). There is a host of non-invasive methods available for registering genital responses (Rodriguez Aranda & Ågmo, 2023; Ventura-Aquino & Ågmo, 2023). Some of these are portable and can be used by participants themselves in their home (Wac, 2009). One excellent example of this is a study by Bloemers et al. (2010) in healthy women and in women diagnosed with hypoactive sexual desire. They were exposed to pornographic video segments while the vaginal and clitoral responses were recorded with photoplethysmography. One session was performed in the lab, and another was performed by the women themselves in their home. To that end, they had been provided with a portable device that transmitted data from the vaginal and clitoral probes to a central database server in real time as well as with a handheld computer controlling the experimental session. That computer administered the video presentations on a laptop screen, among other things. The data from Bloemers et al.’s (2010) study confirmed that the genital response to sexual incentives in the laboratory remains unreduced in women with hypoactive sexual desire, whereas the response at home was much reduced. This elegant experiment not only shows that genital responses can be easily recorded by the participants themselves in their home but also that such recordings may be more sensitive than recordings made in the lab. Moreover, the fact that the genital response was reduced in the home setting but not in the laboratory shows that the diagnosis of hypoactive sexual desire refers to the situational rather than the generalized type. This may be of importance, since most studies of hypoactive sexual desire disorder or sexual arousal/interest disorder fail to make the distinction between the two types. The lack of replication of the Bloemers et al. (2010) study makes the preceding proposals preliminary.

Whereas the genital responses are truly representative of the intensity of sexual motivation, other responses may voluntarily be altered. Among the responses that may be consciously altered are verbal self-reports of own behavior as well as descriptions of states such as desire, satisfaction, etc. Sexual scripts may be a particularly important influence on self-reports. This means that self-reports of the intensity of sexual motivation can be quite different from objective quantifications of the activity in the sexual central motive state. In fact, doubts concerning the validity of self-reports of motivation were expressed long ago in a much-cited paper (McClelland et al., 1989). It has also been pointed out that individuals may be unaware of the existence of a stimulus that importantly influenced a response or even unaware of the existence of the response itself, making self-reports unsuitable whenever unconscious processes are involved (Nisbett & Wilson, 1977).

The uncertainty inherent in self-reports of sexual matters has been confirmed many times. Examples have been given elsewhere (Ågmo & Laan, 2023; Touraille & Ågmo, 2024), but I repeat a few of them here. In one study, men and women completed a series of questionnaires either believing that the experimenter could observe their responses or that they were connected to a lie detector. In the first case, there were large sex differences coinciding with social norms for men and women whereas there was no sex difference in the second condition (Alexander & Fisher, 2003). Likewise, the fixation of gaze on erotic stimuli was much lower in women than in men when they believed that their gaze pattern could be monitored. No such difference was observed when no monitoring took place (Milani et al., 2019). Again, responses were altered according to social expectations.

Estimations of intensity of motives give different results when the estimations are limited to conscious manifestation of motives vs. estimations including unconscious contributions to motivational force (Schultheiss et al., 2009). A meta-analysis showed this to be the case for the motives of achievement, affiliation, and power (Köllner & Schultheiss, 2014). In these motives, it appears that the difference is larger in men than in women. Concerning the sexual motive, the discrepancy between conscious (self-reported) and unconscious (genital response) measures of motivation seems to be larger in women than in men (Chivers et al., 2010).

**Figure 3:**
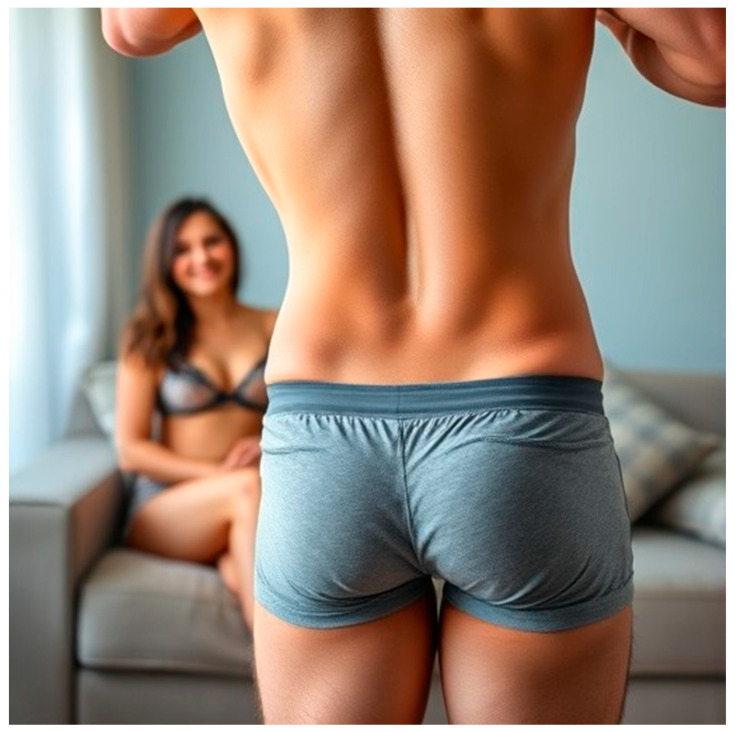
AI generated picture with features similar to those used in the studies of implicit sexual motivation based on the picture story exercise (Hinzmann et al., 2023; Schultheiss et al., 2023). Generously provided by Jessica Hinzmann and Oliver Schultheiss.

Within clinical psychology there has been, and still is, a certain resistance to reducing psychological issues, for example the intensity of sexual motivation, to physiological manifestations. It is believed that there are psychic components that are not appropriately reflected in bodily responses. Consequently, quantification of genital responses would not be a valid representation of the intensity of sexual motivation. Fortunately, there are procedures available for probing the potential psychic components of sexual motivation without encountering the severe deficiencies of self-reports. This may be important if the genital response is not considered to adequately reflect the intensity of sexual motivation. Recently, the picture story exercise, a procedure for estimating implicit motivation, was adapted for estimations of the intensity of sexual motivation (Hinzmann et al., 2023; Schultheiss & Hinzmann, 2025; Schultheiss et al., 2023). Basically, people are exposed to eight ambiguous pictures depicting situations that could lead to sexual interaction while not necessarily doing so. One example is a picture showing a man visible from behind, standing behind a woman and holding the zipper of her dress. Another example shows a woman playing billiards and a man leaning over her, showing her how to handle the cue stick. As an illustration to the kind of motives employed, Figure 3 shows a computer-generated picture preserving the main features of actually used stimuli. Participants are exposed to sexual incentives, e.g., a pornographic video segment, and are then asked to write a story about the pictures. That story is then coded in a standardized way, and an estimate of implicit sexual motivation is obtained. A fundamental assumption behind this kind of procedure is that the unconscious is allowed to influence the story since the individual is unaware of the purpose of the story-writing. Therefore, the censor does not identify and suppress unwanted sexual elements. The access to the unconscious provided by this kind of test was pointed out already in the original description of the forerunner of the present picture story exercises, Murray’s thematic apperception test (Morgan & Murray, 1935). The validity and reliability of the picture story exercise measure have been much discussed, but there are now data showing that both may be acceptable (Lang, 2014; Schultheiss & Köllner, 2021; Schultheiss et al., 2008). They are at least not inferior to what is found for other commonly used tests and questionnaires (McAuliffe et al., 2007; McCrae et al., 2011; Ramjee et al., 1999).

In the specific case of the picture story exercise developed by Schultheiss and colleagues as a test for assessing the need for sex, there is substantial evidence for reliability and causal, indicator, and criterion validity as well as convergent and divergent validity with other implicit and explicit measures (Schultheiss & Hinzmann, 2025). The reliability of picture stories in general has been found to be high (Gruber & Kreuzpointner, 2013; Schultheiss et al., 2008), but no specific data for the need for sex are available. The causal validity was determined by exposing individuals to sexually arousing stimuli after having completed a preexposure picture story exercise. Scores obtained at a postexposure picture story exercise were higher regardless of the kind of sexual stimulus used. There was no difference in need for sex score when the subjects were exposed to neutral or aversive stimuli. Criterion validity was assessed by making the subjects perform a key-press task after exposing them to neutral, sexual, or aversive stimuli. A key press increased the exposure time to pornographic or neutral pictures. After the exposure to sexual stimuli, the subjects chose to prolong the exposure time to pornographic pictures at the cost of reducing exposure time to neutral pictures. Finally, it was found that the need for sex predicted the frequency of sexual activity with a partner, suggesting predictive validity (Schultheiss & Hinzmann, 2025). Even though these data must be considered as preliminary, they suggest that the version of the picture story exercise adapted for assessing the need for sex may be most useful. It may also be interesting to note that there is a tendency to replace motivation with need among those in the field of implicit motivation still adhering to the Murray–McClelland tradition.

There are other procedures for estimating implicit sexual motivation. One is the implicit AMORE (Affective and Motivational Orientation Related to Erotic Arousal Questionnaire) scale (Hill, 2016). Pictures of female–male couples engaged in sexual activities are presented for a short time, followed by an ambiguous object (a character from the Chinese language). The subjects are asked to indicate whether the object was pleasant or unpleasant. Alternatively, subjects can be asked directly to rate the pleasantness of pictures of couples engaged in sexual activity. The test can be performed immediately after exposure to sexual incentives, assuring the presence of an active central motive state. After a series of analyses, it was concluded that the test is a reliable and valid estimator of implicit sexual motivation. Finally, the Implicit Association Test (IAT) originally described by Greenwald et al. (1998) has been adapted for estimating implicit sexual motivation in different contexts (Brand et al., 2024; Dewitte, 2015). Reliability and validity were reported to be satisfactory.

Any of the tests mentioned here, and possibly some other variants of tests for implicit motivation, could be used for evaluating treatment effects on sexual motivation. Thus, there is no contriving reason for not replacing self-reports with a measure of implicit motivation. Thereby, the unconscious contributions to sexual motivation would be included, unmanipulated by volitional activities. Consequently, the possibility of detecting clinically relevant treatment effects would be strongly enhanced. The disadvantage with tests for implicit sexual motivation compared to self-reports is the scoring procedure used in these tests. It is very simple for the latter, and it is easy to computerize the entire scoring procedure. Currently, the scoring of tests for implicit motivation is far more time consuming and cumbersome. Nevertheless, this may very soon become a problem of the past, since modern computing with deep learning algorithms has already been applied to the analysis of texts originating from the picture story exercise (Gruber, 2022; Gruber & Jockisch, 2020; Nilsson et al., 2025; Pang & Ring, 2020; Young, 2024).

In this section, I have shown that there are reliable physiological indicators of the intensity of sexual motivation. Relevant responses can be recorded with non-invasive procedures during exposure to sexual incentives in the participant’s home. Likewise, tests for implicit motivation can be performed immediately after exposure to sexual incentives, thereby allowing for estimation of the intensity of sexual motivation without the intervention of volitional manipulation. By combining these methods, it should be possible to obtain an unbiased measure of potential treatment effects on sexual motivation in men and women.

## 8. Quantification of Sexual Motivation: Non-Human Animals

The contradictory effects of bremelanotide on copulatory behavior in two rodent species mentioned earlier raise serious questions about interspecies generalizability. If results cannot be generalized within the rodent family, how could they be generalized from rodent to human? Furthermore, different presumed indicators of motivation, paracopulatory behaviors vs. sexual reward, show different responses to the same drug. There are several explanations available for this state of affairs. The most obvious is that the behavior pattern chosen as an indicator of the intensity of sexual motivation in some studies (Gelez et al., 2013; Pfaus et al., 2004), the number of stereotyped movements displayed by females during sexual interaction with males, in fact is determined by some factor unrelated to the urge to seek sexual interaction. In fact, none of the behaviors displayed during copulatory interaction are useful for estimating sexual motivation, as pointed out long ago in a seminal contribution (Meyerson & Lindström, 1973). Likewise, the magnitude of reward experienced following sexual interaction may be independent from the motivation to engage in such interaction (Le Moëne & Ågmo, 2019). Moreover, the conditioned place preference procedure employed in the studies mentioned here may not be able to detect differences in magnitude of reward (Huston et al., 2013; Yates, 2023). To determine the level of sexual motivation, defined as the intensity of the urge to seek sexual contact, sexual approach behaviors must be determined (Ventura-Aquino & Ågmo, 2023).

There are currently several procedures available for quantifying sexual approach behaviors in rodents and primates (Ågmo et al., 2004; Avitsur & Yirmiya, 1999; Ventura-Aquino & Paredes, 2017; Ventura-Aquino et al., 2018). Most of them are based on allowing the experimental subjects to choose between a sexually relevant stimulus and a socially relevant stimulus. Typically, a sexually relevant stimulus is a conspecific of the opposite sex whereas a socially relevant stimulus is a conspecific of the same sex or a castrated member of the opposite sex. The larger the difference in approach between these stimuli, the larger the intensity of sexual motivation. The use of this kind of test is increasing, but observations of copulatory behavior are still dominating. Table 1 summarizes the rather limited empirical evidence showing that drug effects on sexual approach behaviors are predictive of effects in the human clinic.

Since the preclinical studies of flibanserin and bremelanotide mentioned earlier failed to include measures of sexual approach, no accurate estimate of the inIttensity of sexual motivation was provided. Consequently, well-founded predictions concerning clinical efficacy could not be made.

The weak generalizability of drug effects on the stereotyped behavior patterns displayed during copulation or on sexual reward has many explanations in addition to unfortunate choice of criterion behavior or test procedure. One is that the neural control of sexual motivation is different in rodents and humans. Another is that human sexual motivation is modulated by factors nonexistent in rodents. In the present communication I have provided evidence in favor of the latter explanation.

## 9. The United States Food and Drug Administration (FDA) Guidelines for Evaluating Drugs for Treating Low Sexual Interest, Desire, and Arousal in Women

To illustrate the practical importance of including non-conscious contributions to sexual motivation in any analysis of the clinical effects of drugs or other treatments, I will critically examine the FDA guidelines, which are strictly based on self-reports. I will even propose that these guidelines may lead to flawed conclusions as to the effectiveness of treatment.

The guidelines suggest that treatment outcomes should be evaluated by determining the change from baseline in the number of satisfying sexual events, the corresponding change in the level of sexual interest or desire, the level of sexual arousal, and the level of distress (Food and Drug Administration, 2016). Sexual interest or desire are determined by questions one and two of the Female Sexual Function Index (Rosen et al., 2000). Question one states “over the past 4 weeks, how often did you feel sexual desire or interest?”. The response options range from 5 (almost always or always) to 1 (almost never or never). Question 2 asks “over the past 4 weeks, how would you rate your level (degree) of sexual desire or interest over the preceding 4 weeks?” Here, the response options range from 5 (very high) to 1 (very low or none at all). Arousal is evaluated in questions three to six in the same questionnaire: (3) Over the past 4 weeks, how often did you feel sexually aroused (“turned on”) during sexual activity or intercourse? Responses range from 0 (no sexual activity) to 5 (almost always or always). (4) Over the past 4 weeks, how would you rate your level of sexual arousal (“turn on”) during sexual activity or intercourse? 0 = no sexual activity; 5 = very high. (5) Over the past 4 weeks, how confident were you about becoming sexually aroused during sexual activity or intercourse? 0 = no sexual activity; 5 = very high confidence. (6) Over the past 4 weeks, how often have you been satisfied with your arousal (excitement) during sexual activity or intercourse? 0 = no sexual activity; 5 = always or almost always. The level of distress is determined with another questionnaire, the Female Sexual Distress Scale–Revised (DeRogatis et al., 2008). Only question 13, “how often did you feel bothered by low sexual desire” is used. Responses can range from 0 (never) to 4 (always).

The FDA guidelines express some concern about the validity and reliability of the questionnaire responses and provide several suggestions as to how improvements could be made. However, none of these suggestions have been implemented in the published clinical studies, neither of flibanserin (Simon et al., 2022) nor of bremelanotide (Kingsberg et al., 2019). It is not only the FDA that has expressed doubts as to the usefulness of the questionnaire responses aimed at determining sexual desire and distress, but also independent scientists (Forbes et al., 2014; Spielmans & Ellefson, 2024). An extensive review found that the Female Sexual Function Index, used as a gold standard for evaluating treatment effects on sexual motivation, has many weaknesses (Neijenhuijs et al., 2019).

Besides the issues about reliability and some types of validity, there is one particularly important issue regarding the FDA guidelines. Since the questionnaires used for evaluation of drug effects on sexual desire, like all other questionnaires based on self-report, tap into recollections of conscious experiences of feeling sexually motivated or aroused, they ignore all unconscious instances of an active sexual central motive state. This might obscure even strong treatment effects on sexual function.

The criticism expressed above may not apply to another end point for drug effects, the number of satisfactory sexual events. It can be expected that these events are conscious experiences. Moreover, since participants in the effect studies are asked to record these events daily, the number should be quite accurate. Whether the events indeed are recorded daily or not is uncertain, however. In the instructions to the participants in the BEGONIA trial, for example, it is stated that “Data could be entered for 7 days after the day in question” (Katz et al., 2013, p. 1809). Neither the proportion of women using the opportunity for delayed reporting neither the length of these delays is reported in the paper. In the other flowery clinical trials of flibanserin (VIOLET (DeRogatis et al., 2012), DAISY (Sand et al., 2010; Thorp et al., 2009), ORCHID (Nappi et al., 2009), and DAHLIA (Clayton et al., 2009; Jayne et al., 2010), the details of the recording of satisfactory sexual events were not even reported. Regardless of this, there are data showing that daily diary self-reports differ substantially from objectively recorded data. This is the case for self-reports of physical activity vs. accelerometric recordings of activity (Prince et al., 2008; Vergauwen et al., 2021), for daily self-reported work performance vs. direct measurements of productivity (Pransky et al., 2006), and for a sleep diary vs. polysomnography (Benz et al., 2023). No comparison between self-reported satisfactory sexual events and objectively recorded sexual activities exists, for obvious reasons, but it may be assumed that self-reports of sexual events are not more reliable than self-reports of physical activity, work performance, or sleep. Thus, even diary reports of satisfactory sex suffer from substantial uncertainty. This means that all end points established for evaluating treatment effects on sexual desire may be seriously deficient.

The obsession with self-reports of different kinds may perhaps be justified by the low cost, both in terms of monetary expenses and in terms of ease and speed of filling out questionnaires. It seems that considerations of this kind have exerted an irresistible attraction.

## 10. Proposals for the Future

In the case of sexual motivation in humans, the genital responses to sexual incentives should be evaluated. Thereby, an objective, physiological measure of the activity in the sexual central motive state, unmodified by volitional events, would be obtained. Some of the methods available for recording genital responses were described earlier. The possibility to objectively quantify these responses in the participants’ home appears to be very attractive. Thereby, questions about external or ecological validity of the measurements are avoided. In addition, the purely mental components of sexual motivation should be assessed with one or several of the tests for implicit sexual motivation. Even though these tests are in their infancy, with data on reliability and validity being limited, they offer invaluable information, impossible to obtain in self-reports. It can be expected that implicit tests will improve because of the increasing acceptance of the contribution of unconscious processes to human behavior and the ensuing increased research on this kind of test.

The sensitivity of the genital response and of tests for implicit sexual motivation is so high that positive effects, or lack thereof, could be reasonably well established already in phase II drug studies, avoiding the onerous costs of phase III studies of drugs already doomed to fail. The remaining problem consists in making the FDA change its unfortunate guidelines. The increase in power of the clinical evaluation obtained by combining objective and implicit measures of sexual motivation would probably be substantial. Being able to reliably estimate the activity in the sexual central motive state, an exquisitely accurate measure of treatment effects, together with elimination of the possibility to willfully manipulate test responses would deliver enormous advantages compared to the present guidelines.

In preclinical studies in animals, sexual approach behaviors should be observed instead of copulatory reflexes. Some of the available procedures were mentioned above. It is most likely that this would enhance the predictive validity and contribute to the streamlining of tests for novel compounds. In principle, genital responses in male and female rodents could also be recorded. Although feasible (Bernabé et al., 1995; Giuliano et al., 1994; Johnson & Murray, 1992; Kitchell et al., 1982), such recordings are time-consuming and invasive. Furthermore, it is not evident that they would provide any information as to the activity in the sexual central motive state not provided by tests for sexual approach. At difference to humans, rodents are unlikely to manipulate their approach behaviors to make them coincide with social expectations or desirability.

With the proposed changes in both the preclinical and clinical testing of new treatments, it is possible that some progress finally could be made in the treatment of dysfunctional sexual motivation.

## 11. Conclusions

Except for psychoanalytic circles, the role of unconscious processes in human sexual motivation has been ignored. With the increased interest in implicit processes, there appears to be an increased acceptance of unconscious contributions to a variety of human behaviors. In the case of sex, expressions and the conscious experience of sexual motivation are heavily influenced by acquired sexual scripts. Volitional alterations in the manifestations of sexual motivation are therefore common and may obscure the underlying processes. Therefore, it is important to find manifestations of sexual motivation that are not consciously altered to conform to social expectations. Self-reports are always subjected to such alterations and are consequently unsuitable for obtaining unbiased estimates of the intensity of sexual motivation. Measures unaltered by volitions, like genital responses and tests for implicit sexual motivation, avoid this problem.

The adoption of methods assuring reliable and unbiased estimations of sexual motivation in humans, including the unconscious contributions, might allow for the discovery of new and efficient treatments of sexual dysfunction, particularly those related to sexual desire. To continue to ignore the unconscious parts of sexual motivation would, to the contrary, impede progress in the search for such treatments. In the case of preclinical studies, it would be desirable to abandon the habit of studying the reflexive components of copulatory interaction in rodents and replace it with studies of sexual approach. The cost, in terms of quality of life, of persisting with deficient preclinical and clinical evaluation of drugs for treating dysfunctions of sexual motivation may be considerable for those suffering from such dysfunctions. A paradigm change, both in preclinical and clinical research, is needed to avoid these costs.

## Figures and Tables

**Table 1:** Drug effects on rodent copulatory and sexual approach behaviors compared to known effects in humans.

Drug	Test	Effect Rat	Clinical Effect Human
Paroxetine ^a^	Paced mating	None ^1^	Enhanced incidence of sexual dysfunctions including reduced motivation ^2^
	Sexual approach	Reduced ^3^	
Fluoxetine ^b^	Copulatory behavior	None ^4^ or enhanced ejaculation latency ^5^ reduced lordosis ^6^	Enhanced incidence of sexual dysfunctions perhaps even reduced motivation ^7^
	Sexual approach	Reduced ^8^	
Yohimbine ^b^	Sexual approach	Enhanced ^9^	Possibly increased sexual motivation ^10^

The list of drugs is very short, since few drugs have been evaluated both in rodent sexual approach and in the human clinic. The paced mating test is a procedure in which the female controls the sexual interaction. This is achieved by dividing the observation arena with a barrier containing at least one opening, large enough for allowing passage of the thin female but too small for the bigger male. Thus, the female can move freely within the arena while the male is confined to one part. The female controls the pace of sexual interaction. In tests for copulatory behavior, one male and one female are placed together without opportunity for escape. Sexual approach tests are described above. The superscript numbers refer to supporting references. Those given are only examples. ^a^, references refer to female rats only. ^b^, references refer to both male and female rats. ^1^ (Kaspersen & Ågmo, 2012; Snoeren et al., 2011a, 2011b). ^2^ (Higgins et al., 2010; Rosen et al., 1999). ^3^ (Kaspersen & Ågmo, 2012). ^4^ (Le Moëne & Ågmo, 2019). ^5^ (Hueletl-Soto et al., 2012). ^6^ (Maswood et al., 2008). ^7^ (Higgins et al., 2010; Rosen et al., 1999). ^8^ (Matuszczyk et al., 1998; Uphouse et al., 2015). ^9^ (Viitamaa et al., 2006). ^10^ (Rowland et al., 1997; Tam et al., 2001).

## Data Availability

No dataset was generated or analyzed in the course of the present study.
